# Modeling of Electric Fields in Individual Imaging Atlas for Capsular Threshold Prediction of Deep Brain Stimulation in Parkinson's Disease: A Pilot Study

**DOI:** 10.3389/fneur.2020.00532

**Published:** 2020-07-02

**Authors:** Matthieu Béreau, Astrid Kibleur, Walid Bouthour, Emilie Tomkova Chaoui, Nicholas Maling, T. A. Khoa Nguyen, Shahan Momjian, Maria Isabel Vargas Gomez, André Zacharia, Julien F. Bally, Vanessa Fleury, Laurent Tatu, Pierre R. Burkhard, Paul Krack

**Affiliations:** ^1^Department of Neurology, Besançon University Hospital, Besançon, France; ^2^Department of Neurology, Geneva University Hospital, Geneva, Switzerland; ^3^Boston Scientific Corporation, Valencia, CA, United States; ^4^Department of Neurology, Bern University Hospital, Bern, Switzerland; ^5^Department of Neurosurgery, Geneva University Hospital, Geneva, Switzerland; ^6^Department of Neuroradiology, Geneva University Hospital, Geneva, Switzerland

**Keywords:** Parkinson's disease, deep brain stimulation, subthalamic nucleus, capsular prediction, volume of tissue activated

## Abstract

**Background:** Modeling of deep brain stimulation electric fields and anatomy-based software might improve post-operative management of patients with Parkinson's disease (PD) who have benefitted from subthalamic nucleus deep brain stimulation (STN-DBS).

**Objective:** We compared clinical and software-guided determination of the thresholds for current diffusion to the pyramidal tract, the most frequent limiting side effect in post-operative management of STN-DBS PD patients.

**Methods:** We assessed monopolar reviews in 16 consecutive STN-DBS PD patients and retrospectively compared clinical capsular thresholds, which had been assessed according to standard clinical practice, to those predicted by volume of tissue activated (VTA) model software. All the modeling steps were performed blinded from patients' clinical evaluations.

**Results:** At the group level, we found a significant correlation (*p* = 0.0001) when performing statistical analysis on the *z*-scored capsular thresholds, but with a low regression coefficient (*r* = 0.2445). When considering intra-patient analysis, we found significant correlations (*p* < 0.05) between capsular threshold as modeled with the software and capsular threshold as determined clinically in five patients (31.2%).

**Conclusions:** In this pilot study, the VTA model software was of limited assistance in identifying capsular thresholds for the whole cohort due to a large inter-patient variability. Clinical testing remains the gold standard in selecting stimulation parameters for STN-DBS in PD.

## Introduction

**Figure 1:**
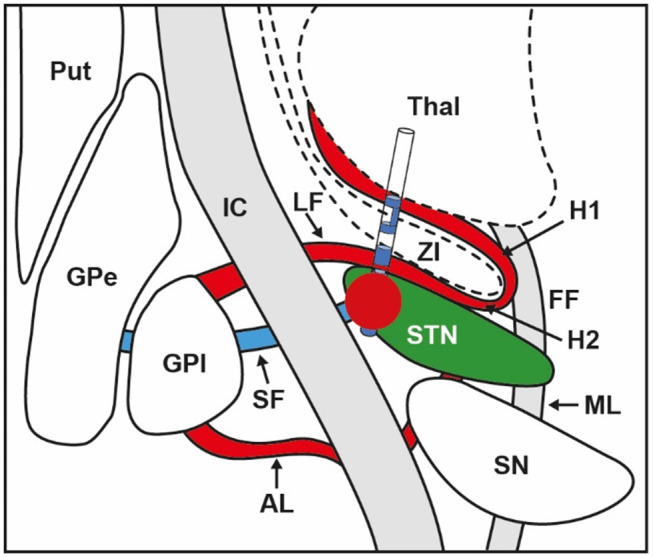
Anatomical relations between electrode position within the STN, volume of tissue activated (VTA) illustrated by the red bubble, and surrounding structures. STN, subthalamic nucleus; VTA, volume of tissue activated; GPi, globus pallidus internus; GPe, globus pallidus externus; Put, putamen; SN, substantia nigra; Thal, thalamus; IC, internal capsule; SF, subthalamic fasciculus; AL, ansa lenticularis; LF, lenticularis fasciculus; ZI, zona incerta; ML, medial lemniscus; FF, fields of Forel; H1, H1 field of Forel; H2, H2 field of Forel. Adapted from Hamani et al. (13).

Deep brain stimulation (DBS) of the subthalamic nucleus (STN) is a well-established treatment for advanced Parkinson's disease (PD) (1). Numerous prospective and open-label studies have demonstrated benefits of DBS for motor and non-motor signs, as well as improved quality of life in PD (2–6). Initial DBS programming is based on establishing and ranking contacts that exhibit the larger therapeutic window, calculated as the difference between stimulation amplitudes at persistent side effects and meaningful improvement of rigidity (7). Side effects are either related to stimulation of the sensorimotor part of the STN on its own, or to current diffusion to neighboring structures including limbic or cognitive parts of the STN, internal capsule (IC), pallidothalamic tract, as well as the cerebellothalamic tract (8–10). Thus, outcome greatly depends on the electrode position and precise knowledge of the surrounding anatomy (11, 12). The most prominent side effect is linked to current diffusion to the corticospinal and corticonuclear tracts (CSNT) within the IC [see Figure 1, adapted from Hamani et al. (13)]. Current diffusion to the CSNT induces tonic muscle contractions, mainly in the face area and fine muscles of the hand, and less frequently in the lower limbs (14), likely reflecting somatotopic arrangement of CSNT fibers in the vicinity of the STN. Dysarthria is related to current diffusion to corticonuclear fibers that innervate muscles involved in speech, i.e., lips, tongue, pharynx, and larynx muscles (8, 14, 15). Although basic algorithms for programming and troubleshooting sessions have been continuously refined, computational modeling of DBS electric fields may potentially provide new information that could be of further aid in DBS programming sessions (16). These models allow calculation of the volume of tissue activated (VTA), for instance, using a diffusion tensor-based finite element neurostimulation model (17). Recently, new directional electrodes have been developed according to multiple independent current source control technology (18, 19). As opposed to conventional electrodes, directional electrodes are characterized by the ability to steer current toward three distinct directions, not only along the *Z*-axis (*Z*), but also in the horizontal plane (*X, Y*) (18, 19). While conventional electrodes generate a spherical electrical field that encompasses all adjacent structures equally, directional contacts produce a limited and adaptable electrical field biased toward the active contact (18, 19). Few studies have outlined the potential interest of directional electrodes for patient management (7, 20, 21). Importantly, the number of individual stimulation parameter combinations increases substantially with the use of directional electrodes, making post-operative management more challenging and time-consuming. Thus, clinical tools including VTA models could be helpful in identifying effective parameter settings for each patient more easily. The goal of this pilot study was to evaluate the usefulness of a new software designed for clinical practice in the refinement of DBS parameters.

## Materials and Methods

### Population

We enrolled 16 STN-DBS PD patients. All patients were implanted with Cartesia directional devices (Boston Scientific, Valencia, CA) (20, 22) that each contained one ventral and one dorsal non-segmented ring (rings 1, 8 and 9, 16 for left and right STN, respectively), and two segmented rings in between (one ventral and one dorsal segmented ring), each containing three contacts (contacts 2, 3, 4 and 5, 6, 7 for left STN ventral and dorsal segmented rings, and contacts 10, 11, 12 and 13, 14, 15 for right STN ventral and dorsal segmented rings, respectively).

### Clinical Evaluation

We assessed monopolar reviews according to standard clinical practice during one session after a 12-h overnight withdrawal from dopaminergic medication as described previously (23). We tested each electrode separately and kept the contralateral STN-DBS ON for the patient's comfort. We predefined a frequency of 130 Hz and pulse width of 60 μs for all sessions. We determined the capsular threshold amplitude (CTA) by testing for each side the two non-segmented rings, the two segmented rings in between on omnidirectional stimulation (vertical steering), as well as the six directional contacts from the two segmented rings (horizontal steering), which added up to a total of 10 measures per electrode. We gradually increased current amplitude by steps of 0.5 mA until the appearance of a visible facial or limb contraction. Then, we decreased the current amplitude in steps of 0.1 mA until the exact contraction threshold was reached (23).

### Capsular Threshold Modeling

We used the Guide™ XT software for all steps detailed in this section. MB performed manual refinement of the IC volume and AK performed capsular threshold determination on the images using the VTA. We carried out all the modeling procedures blinded from patients' clinical evaluations, with anonymization of the patient's name on the MRI images. We first performed coregistration of the preoperative 3D T1, specific STN visualization T2 MRI sequences (3D T2 SPACE in sagittal orientation, FOV 450 mm, TR/TE 2400/225 ms, flip angle 120°), and the post-operative CT scan. Then, an automatic anatomical segmentation of the STN and the IC was computed. For each patient, we manually refined the IC using both the T2 image and the Schaltenbrand atlas (24), in order to extend IC boundaries to the cerebral peduncles in the midbrain, a process that enables visualization of the CSNT in the vicinity of the STN. Manual refinement of the IC was the longest step of the process, and took 10–15 min per patient. Lead trajectories were automatically reconstructed from the CT scans. We subsequently adjusted the lead orientations determined from the post-operative sagittal and coronal topograms using the radiopaque marker embedded in each lead. Finally, we sequentially built VTAs by increasing the current amplitude virtually for each contact tested until the VTA border touched the capsule border. We labeled the corresponding amplitude as the CTA of the VTA model (VTA-CTA).

### Statistics

For each patient, we computed statistical analysis on the data to test the correlation between the clinical thresholds and the modeled capsular threshold. We used the corrcoef function of the Matlab (Mathworks, Natick, MA) statistical toolbox. We also performed a correlation analysis on the whole group and normalized the data to compare patients by using the *z*-scores. We then reported the resulting *p* and correlation coefficients *r* for each patient. Lastly, we refined the analysis for the patients who did not show any significant effect by separating the analysis for the left and right hemispheres to see if the effect had been hidden by one side where the modeling predicted the capsular threshold poorly.

## Results

### Population

We tested 29/32 STNs from 16 consecutive patients (6 women and 10 men). Three STNs were ruled out given that no capsular side effects were observed between 0 and 6 mA, and CTA was not determined. Mean patient age was 60.1 ± 2.0 years, and mean PD duration was 10.5 ± 1.2 years. Mean presurgical MDS-UPDRS part III scores in off and on drug conditions were 47.1 ± 4.0 and 14.3 ± 2.0, respectively, corresponding to a mean improvement of 70.2 ± 3.5%. Monopolar reviews took place between 2.5 and 6 months after surgery (mean, 3.7 ± 0.2), and mean levodopa equivalent daily dose (LEDD) before and after DBS were 1182.8 ± 115.8 mg and 452.6 ± 88.4 mg, respectively, corresponding to a mean reduction of dopaminergic therapy dosage of 60.9 ± 7.5% (25).

### Clinical Threshold vs. Capsular Threshold

**Figure 2:**
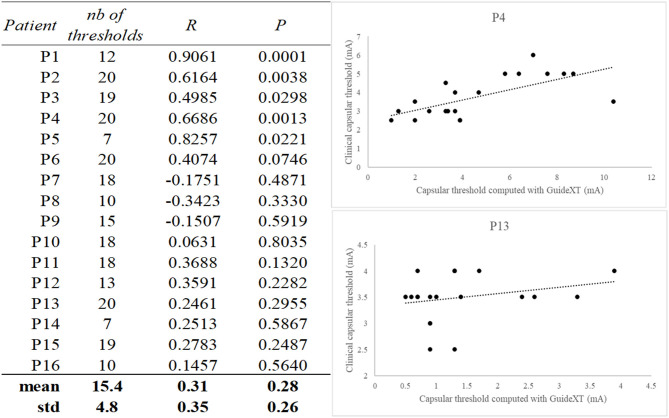
Table of correlation coefficients and associated *p*-values between Guide™ XT predicted capsular threshold and clinically measured threshold. Examples of scatterplots from one good correlation (P4) and one bad correlation result (P13). The number of calculated capsular thresholds per patients is indicated in the first column.

At the group level, when performing statistical analysis on the *z*-scored capsular thresholds, we found a significant correlation (*p* = 0.0001), with a relatively low regression coefficient (*r* = 0.2445). When considering intra-patient analysis, we found significant (*p* < 0.05) correlations between capsular threshold as modeled with the software and capsular threshold as determined clinically in five patients (see Figure 2). In these five patients, the resulting mean correlation coefficient was 0.70 (±0.16). Furthermore, in one additional patient, there was a tendency toward significant correlation (*p* = 0.0746).

### Vertical and Horizontal Virtual Current Steering

**Figure 3:**
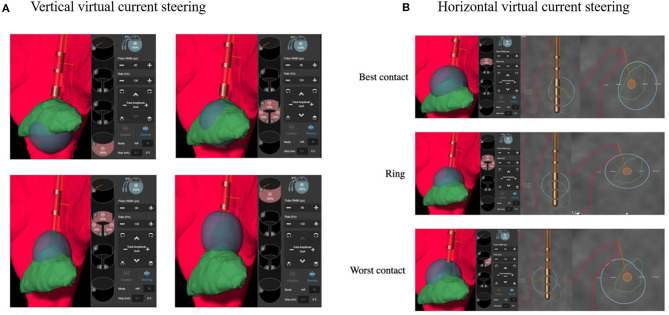
Example of vertical **(A)** and horizontal **(B)** virtual current steering with Guide™ XT. Vertical virtual current steering **(A)** shows that contact 1 is within the ventral part of the STN, and contact 8 is outside the STN. Ventral (2–4) and dorsal (5–7) rings are within the ventromedial and dorsolateral parts of the STN, respectively. According to horizontal virtual current steering **(B)**, modeled VTA of contact 7, “the best contact,” does not touch the CSNT, whereas modeled VTA of either the ring or contact 6, “the worst contact,” shows current diffusion touching the CSNT. VTA (in blue): volume of tissue activated modeled by Guide™ XT, STN (in green): subthalamic nucleus automatically segmented by Guide™ XT, internal capsule (in red) including CSNT manually refined: corticospinal and corticonuclear tracts.

We analyzed virtual current steering in patients for whom CTA was correctly predicted by the VTA-CTA model. We arbitrarily chose the constant parameter settings as follows: 3 mA, 60 μs, and 130 Hz. Then, we built a VTA according to the software and successively considered the vertical and horizontal virtual current steering (see Figure 3). Vertical virtual current steering consisted of representing the lead position and overlap between the VTA and STN automatically segmented by the software. Horizontal virtual current steering consisted of representing the lead position and overlap between VTA, STN, and IC in the best ring previously identified, in order to rank its contacts from lowest to highest capsular threshold.

## Discussion

In this pilot study, we tested the hypothesis that VTA model software could help clinicians with capsular threshold determination in STN-DBS PD patients during post-operative management. To do this, we retrospectively compared predicted capsular thresholds modeled using the software with clinical capsular thresholds clinically assessed during the monopolar review in 16 STN-DBS PD patients.

At the group level, we found a significant correlation (*p* = 0.0001), between image-defined and clinically defined capsular thresholds, but the regression coefficient was relatively low (*r* = 0.2445 points). Intra-patient analyses showed significant (*p* < 0.05) correlations in only 5/16 patients (31.2%), meaning that for each of these patients, contacts were accurately ranked by the software from the lowest to the highest capsular threshold. The low regression coefficient in analysis at the group level can therefore be explained by some variability of the tool as confirmed in intra-patient analyses, with an excellent matching in some, but not all, of the patients.

Although the small size of this pilot study, a lack of statistical power, and a learning curve effect for both clinical and VTA-modeled capsular threshold measurements must be mentioned first, factors related to the different steps needed for capsular threshold modeling may be considered one by one to explain the discrepancy observed between global and intra-patient analyses.

Coregistration between preoperative 3D T1, specific STN visualization T2 MRI sequences, and the post-operative CT scan was performed automatically by the software. Visual inspection of coregistration quality was systematically performed before anatomical segmentation. Coregistration was correctly done in all cases and does not appear to be a critical factor contributing to the variability observed between patients.

Anatomical segmentation in the software does not include the CSNT *per se* but the IC, which does not integrate the diencephalic–mesencephalic junction. Thus, in order to determine the VTA-modeled capsular threshold, we manually drew the CSNT in the vicinity of the STN, by extending the automatically segmented IC from the diencephalon to the mesencephalon. Nevertheless, this step, the longest of the capsular threshold modeling, was not highly reproducible, which might broadly explain the variability observed from one patient to another. One would expect that capsular threshold as modeled with the software depends on the distance between the internal border of the CSNT drawn manually and the lead where the VTA is built. In the near future, tractography could be a great help to reduce this bias (24–27).

Determination of the electrode position from the post-operative CT scan and coregistration of the CT with the T1 pre-operative MRI is also associated with some intrinsic limitations of the software, namely, electrode position reconstruction from the CT artifact, brainshift pre- and post-implantation, and T1 image artifacts.

Lead orientations that have been performed manually from the topograms (sagittal and coronal views) may also constitute a source of error in the modeling as they can change the determination of the capsular threshold between the three contacts at each depth of directional contact. Methods such as 3D rotational fluoroscopy and CT scan-based algorithms have been developed to determine orientation automatically with higher precision and less variability, but had not been included in the software at the time of the study (28, 29).

The VTA model used by the software for capsular threshold modeling is a critical point to discuss. The model makes several assumptions that influence its threshold estimates. The first is a simplified electrical medium that represents the brain as a homogenous and isotropic volume conductor. This means that every point in the model has the same electrical conductivity value (homogeneous) and that those conductivity values are the same in every direction (isotropic). Heterogenous and anisotropic estimates of tissue conductivity are an area of ongoing research (30). Furthermore, we do not know the amplitude range for which the model is valid (power dispersion in biological anisotropic tissue). Additionally, all axons in the model are assumed to be equal in diameter, perfectly straight, and running perpendicular to the trajectory of the lead. The diameter of an axon is known to strongly influence its excitability (31), and the IC exhibits axons with a range of diameters (32). Furthermore, the trajectory and orientation of the axons also influence the excitability of the model. These model assumptions were instituted both to simplify the model and to tend toward the side of excitability.

Despite the multiple sources of error and current limitations discussed above, the software could offer the opportunity to obtain a more comprehensive anatomy-based approach for directional DBS and potentially less time-consuming bedside management for a given patient. Although clinical capsular threshold determination remains the gold standard, VTA model software could be a useful tool for the refinement of parameter settings in patients for whom the clinical threshold and VTA modeled threshold are congruent (see Figure 3). Vertical virtual current steering, which consists of arbitrarily choosing constant parameter settings, for instance: 3 mA, 60 μs, 130 Hz, and drawing the VTA for the four electrode levels may help clinicians to visualize the lead position within the STN and the overlap between the VTA and the sensorimotor part of the STN. This virtual approach may help to more rapidly determine the best anatomical match out of four levels for stimulation from ventral to dorsal, targeting the sensorimotor STN for optimal improvement in parkinsonism. Horizontal virtual current steering, which consists of arbitrarily choosing constant parameter settings, for instance: 3 mA, 60 μs, 130 Hz, and drawing the VTA for the three contacts contained in one segmented ring, could illustrate overlap between the VTA, STN, and IC in order to rank contacts from the lowest to the highest capsular threshold. Such information could be valuable in determining visually guided and anatomically based horizontal steering strategies, when stimulation is limited by side effects related to current diffusion to surrounding fiber systems such as the CSNT. This approach could also be extended to other DBS side effects such as paresthesia, which is related to current diffusion to the medial lemniscus.

## Conclusion

In this pilot study, software that superimposes VTA and anatomy was of limited assistance in the identification of capsular thresholds for the whole cohort due to large inter-patient variability. So far, this new tool allows visualization of the IC, but it has not been designed to identify corticonuclear and corticobulbar tracts inside this very large structure. Integration of fiber tracking tools that can visualize these fiber systems might lead to a better match between visual modeling and clinical testing, which would be an important first step to a more automatized post-operative management of DBS. Currently, clinical testing remains the gold standard in selecting stimulation parameters for STN-DBS in PD. Further studies with larger sample sizes remain mandatory to assess the usefulness of VTA model software for practical management of STN-DBS PD patients.

## Data Availability Statement

The datasets generated for this study are available on request to the corresponding author.

## Ethics Statement

Ethical approval was not required as per local legislation and national guidelines. The patients/participants [legal guardian/next of kin] provided written informed consent to participate in this study.

## Author Contributions

MB, AK, WB, PK, and PB: conception and organization of the research project. MB, AK, WB, ET, and AZ: execution of the research project. MB, AK, and WB: writing of the first draft of the manuscript. ET, NM, TN, SM, MV, AZ, JB, LT, PB, and PK: review and critique of the manuscript. All authors: contributed to the article and approved the submitted version.

## Conflict of Interest

MB, WB, and SM received travel and accommodation funding from Boston Scientific unrelated to the research covered in this article. PK reports grants and fees for lecturing from Boston Scientific (paid to employer) and grants from Swiss National Science Foundation, ROGER DE SPOELBERCH Foundation, Bertarelli Foundation, Annemarie Opprecht Foundation, Parkinson Schweiz, and the Michael J. Fox Foundation, outside the submitted work. The remaining authors declare that the research was conducted in the absence of any commercial or financial relationships that could be construed as a potential conflict of interest.

## Abbreviations

STN: subthalamic nucleus
DBS: deep brain stimulation
VTA: volume of tissue activated
CTA: capsular threshold amplitude
VTA-CTA: VTA modeled capsular threshold amplitude
IC: internal capsule
CSNT: corticospinal and corticonuclear tracts.
